# The relationship between dual-task and cognitive performance among elderly participants who exercise regularly

**DOI:** 10.1590/bjpt-rbf.2014.0082

**Published:** 2015-04-27

**Authors:** Luciana C. A. Lima, Juliana H. Ansai, Larissa P. Andrade, Anielle C. M. Takahashi

**Affiliations:** Departamento de Fisioterapia, Universidade Federal de São Carlos (UFSCar), São Carlos, SP, Brazil

**Keywords:** cognition, aging, dual task, gait, physical exercise, physical therapy

## Abstract

**BACKGROUND:**

The dual-task performance is associated with the functionality of the elderly and it becomes more complex with age.

**OBJECTIVE:**

To investigate the relationship between the Timed Up and Go dual task (TUG-DT) and cognitive tests among elderly participants who exercise regularly.

**METHOD:**

This study examined 98 non-institutionalized people over 60 years old who exercised regularly. Participants were assessed using the TUG-DT (i.e. doing the TUG while listing the days of the week in reverse order), the Montreal Cognitive Assessment (MoCA), the Clock Drawing Test (CDT), and the Mini Mental State Examination (MMSE). The motor (i.e. time and number of steps) and cognitive (i.e. number of correct words) data were collected from TUG-DT . We used a significance level of α=0.05 and SPSS 17.0 for all data analyses.

**RESULTS:**

This current elderly sample featured a predominance of women (69.4%) who were highly educated (median=10 years of education) compared to Brazilian population and mostly non-fallers (86.7%). The volunteers showed a good performance on the TUG-DT and the other cognitive tests, except the MoCA, with scores below the cutoff of 26 points. Significant and weak correlations were observed between the TUG-DT (time) and the visuo-spatial/executive domain of the MoCA and the MMSE. The cognitive component of the TUG-DT showed strong correlations between the total MoCA performance score and its visuo-spatial/executive domain.

**CONCLUSIONS:**

The use of the TUG-DT to assess cognition is promising; however, the use of more challenging cognitive tasks should be considered when the study population has a high level of education.

## Introduction

According to the Brazilian Institute of Geography and Statistics, the proportion of individuals older than 65 years old, which represented 5.9% of the country's population in 2000, will increase to 22.71% by 2050^1^. Aging is associated with reductions in cognitive and (consequently) physical functioning as well as increases in dependence and the risk of falls^2^.

Cognitive screening enables the prevention, identification, diagnosis, and timely treatment of early cognitive decline among older adults. Furthermore, this screening enables practitioners to monitor small changes in cognitive function^2^^-^ 4. The Mini Mental State Examination (MMSE), the Montreal Cognitive Assessment (MoCA), and the Clock Drawing Test (CDT)^5^^,^ 6 are some of the tests used for cognitive screening. Although these tests are simple and easy to apply, they do not represent functional activities among older adults, and they are significantly influenced by the respondents' educational level.

Among older adults, cognitive deficits (especially executive function impairments) likely interfere with balance and gait. The automatic control of walking is impaired with age, and walking becomes an activity that demands attention and is therefore controlled by a cortical region^7^. Many instances of falls occur when older adults attempt to walk and perform a secondary task at the same time. Dual-task performance is defined as the simultaneous execution of a primary and a secondary task (e.g., walking and talking). Dual-task performance is common in the everyday lives of the elderly. Between-task competition and the decline in the executive capacity associated with aging hinder dual-task performance among older adults^8^^-^ 10.

The Timed Up and Go (TUG) task is a reliable and easy-to-apply assessment of mobility in terms of the risk of falls; the TUG dual task (TUG-DT) is increasingly used to assess the functionality of older adults attempting to combine a motor and cognitive task^11^^-^ 13. Barbosa et al.^12^ found that healthy older adults performed more poorly on the TUG-DT than the TUG, regardless of the type of secondary task. In addition, their performance was especially poor on the more complex secondary tasks such as reciting the days of the week in reverse order^12^.

Dual-task studies have been conducted among individuals with Parkinson's disease^14^, older adults diagnosed with depression^15^, pre-frail older adults^16^, and individuals with Alzheimer's disease^17^. However, no study has investigated the efficacy of the TUG-DT as a cognitive screening tool among older adults who exercise.

Therefore, the present study investigated the relationships between the TUG-DT and the MoCA, CDT, and MMSE among older adults who exercise and live in the community. We hypothesized that the TUG-DT and the cognitive tests would be correlated, thereby indicating the possible effectiveness of the TUG-DT as a quick, practical, and inexpensive screening tool in clinical practice for the early detection of cognitive dysfunctions among older adults who exercise.

## Method

The research ethics committee of the *Universidade Federal de São Carlos* (UFSCar), São Carlos, SP, Brazil approved the present cross-sectional correlation study (ruling no. 297,777/2013). All of the participants signed an informed consent document in compliance with the recommendations of National Health Council resolution no. 196/96(4)^18^.

### Participants

Subjects were recruited from the Adult Revitalization Program (*Programa de Revitalização de Adultos*) and the Servant Quality of Life Program (*Programa de Qualidade de Vida dos Servidores*) of UFSCar.

To be included in this study, subjects had to be over 60 years old, living in the community, able to walk alone without any type of walking aid, and have exercised 50 minutes at least three times per week over at least 1 year. Subjects were excluded for failure to perform the three scheduled cognitive assessments or having diagnoses of Parkinson's, dementia, or stroke with motor sequelae.

The sample size was calculated using G*Power 3.1 based on 1) the study design; 2) a type I error rate of 5% (α=0.05); 3) a statistical power of 80% (1-β=0.80); and 4) a moderate effect size. The total estimated sample included 82 subjects.

### Assessment

All 82 subjects were assessed on the same day. The assessments included the following sequence of items: a clinical interview; a physical activity level evaluation using the Modified Baecke Questionnaire for Older Adults (MBQOA); the number of falls over the last 3 months were recorded; and the TUG, the TUG-DT, cognitive tests (MoCA, MMSE, and CDT) were administered.

The MBQOA is a simple and easy-to-apply questionnaire that assesses level of physical activity over the past 12 months via questions concerning sports, leisure, and household activities. This questionnaire has been validated for use among older Brazilian adults^19^. The MBQOA has no maximum score but higher scores denoted higher levels of physical activity. The MBQOA cutoff score of 3.19 was used to distinguish between sedentary and non-sedentary older adults^20^.

Participants reported the number of times that they fell over the past 3 months. A fall was defined as "an event that results in a person coming to rest inadvertently on the ground or other lower level, other than as a consequence of a violent blow, loss of consciousness, sudden onset of paralysis or an epileptic seizure"^21^.

The TUG was performed with a 45-cm high chair with a back and armrests. In this test, participants rise from the chair following the command "go" by using the arms for support, walk 3 meters at their usual speed, return to the chair, and sit down. The time needed to complete this task was measured using a chronometer. At the starting point, participants leaned back in the chair. The task duration was measured as up to the moment when participants leaned back in the chair again. The cutoff point to define the risk of falls inthe TUG among older Brazilian adults was 12.47 seconds^22^.

In TUG-DT , participants completed a cognitive task (i.e., recited the days of the week in reverse order starting from Sunday) while performing the TUG. The participants did not stop walking even when they made a mistake or forgot the days of the week. The task of reciting the days of the week in reverse order was selected because Barbosa et al.^12^ stated that it was the most challenging and complex task for older adults. The number of words, hits (H), errors (E), words per unit of time (words/time), hits per unit of time (H/time), number of word errors, (E/(H+E)), and number of steps were analyzed. One step was defined as from the moment the heel lost contact to the ground until it touched the ground again. In addition, dual-task cost was calculated by dividing the difference between the time required to complete TUG and TUG-DT by the time required to complete TUG, expressed as a percentage. A positive cost indicates poorer dual-task performance.



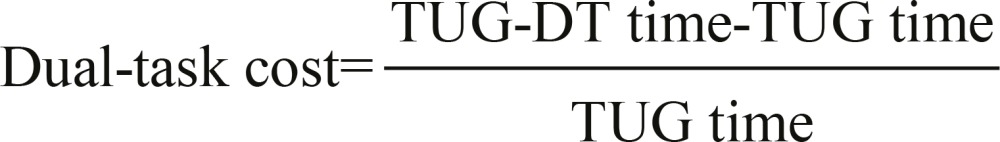



Two examiners conducted the TUG-DT: one recorded the task time and counted the number of steps required to complete it, while the other registered the number of hits and errors. To confirm the numbers of hits and errors, a tape recorder was used throughout the assessment. A pretest was performed to allow the participants to acquaint themselves with the TUG-DT^14^^,^ 23. The TUG-DT was performed first; then, the volunteers completed the MoCA and MMSE.

The MoCA was formulated to detect mild cognitive impairment and mild dementia, and it has been validated for the Brazilian population^24^. The MoCA considers several cognitive abilities including visuospatial/executive function, naming, attention, language, abstraction, delayed recall, and orientation. According to its validation, one point is added to the total score for participants with 12 years of formal education or less. The maximum score is 30; scores 26 and above are considered to indicate the absence of severe cognitive impairment^24^. Although the total score is the most widely used to screen for mild cognitive impairments, the present study also analyzed the MoCA domains separately.

In addition, the MMSE has been validated to assess cognitive functioning among the Brazilian population^25^. The MMSE is comprised of the following cognitive domains: orientation, registration, attention and calculation, recall, and language. In the present study, only the total score was used, which can vary from 0 to 30. The cutoff points for the Brazilian population are as follows: no formal education: 20; 1 to 4 years of formal education: 25; 5 to 8 years of formal education: 26.5; 9 to 11 years of formal education: 28; and over 11 years of formal education: 29^25^.

To perform the CDT, the participants drew a clock representing 2:45. The results were analyzed following Sunderland et al.'s^26^ criteria that consider the clock's contours as well as the placement of the numbers and hands. The CDT score varies from 1 to 10. Scores of 6 or less denote very poor performance, and scores 9 and 10 indicate normal performance among older adults^26^.

The cognitive tests were applied in a room that was as free from visual and auditory stimuli as possible. The examiners were trained to apply the tests before the testing was begun.

### Statistical analysis

Descriptive analyses of the data and point-and-interval estimations of the parameters of interest were performed. The significance level was set as α=0.05. The statistical analyses were performed using SPSS 17.0.

The data distribution was assessed first using the Kolmogorov-Smirnov normality test. Because the data were not normally distributed, Spearman's correlation was used to analyze the relationships between the TUG-DT score and the MoCA (i.e. total score and individual domain scores), MMSE (total score), and the CDT. The strength of the correlation was categorized using Munro's categories^27^, in which weak=0.26-0.49, moderate=0.50-0.69, strong=0.70-0.89, and very strong=0.90-1.00.

## Results

The sample was composed of 98 participants (median=68 years old), most of whom were female (69.4%) non-fallers (86.7%) with a high level of education (median=10 years). The participants did not engage in polypharmacy, and 81.6% did not use psychotropic medications. The median body mass index was 27.23 kg/m², which is slightly above the normal weight for their age^28^. The median MBQOA score was 4.9, denoting a non-sedentary population (Table 1).

**Table 1.** Sociodemographic and clinical characteristics of the 98 Subjects.

Characteristics	M (IQR) or n (%)
Age (years)	68 (64-74)
Females	68 (69.4%)
Falls over the past 3 months	13 (13.3%)
Education (years)	10 (4-16)
BMI	27.23 (23.88-29.65)
Number of medications in use	2 (1-3)
Use of psychotropic drugs	18 (18.4%)
MBQ Score	4.90 (3.80-7.33)

M (IQR): Median (interquartile range); BMI (kg/m2): body mass index; MBQ: Modified Baecke Questionnaire For Older Adults.

Table 2 describes the results of the TUG-DT and the cognitive tests. Overall, the participants exhibited satisfactory performance on the TUG-DT because the median number of errors was zero. The median scores on the CDT, MMSE, and MoCA total score were 9, 28, and 22, respectively. The MoCA domains showing the best performance were naming (median score/maximum possible score: 3/3) and orientation (6/6). Those showing the poorest performance were recall (3/5), abstraction (1/2), language (1/3), and visuospatial/executive function (3/5).

**Table 2.** Cognitive test performance of 98 subjects.

Tests	M (IQR)
TUG-DT Scores	
Number of steps	13.5 (12-15)
Time (s)	9.56 (8.09-12.01)
Task cost (%)	11.41 (1.83-30.99)
Hits	7.00 (6-10)
Errors	0 (0-0)
E/(H+E)	0 (0-0)
H/time	0.81 (0.61-1.01)
Words/time	0.85 (0.68-1.13)
MoCA Scores	
Total score (0-30)	22 (20 - 25)
Visuospatial/executive (0-5)	3 (2 - 4)
Naming (0-3)	3 (2 - 3)
Attention (0-6)	5 (4 - 6)
Language (0-3)	1 (1 - 2)
Abstraction (0-2)	1 (0 - 1)
Delayed recall (0-5)	3 (2 - 4)
Orientation (0-6)	6 (6 - 6)
MMSE Score	63.3/100%^*^
Total score (0-30)	28 (26 - 29)
CDT Score (0-10)	9 (7.75 - 9)

M (IQR): Median (interquartile range); TUG-DT: Timed Up and Go test with dual task; E/(H+E) number of word errors per total words; H/time: hits per unit of time; Words/time: number of words per unit of time; MoCA: Montreal Cognitive Assessment; MMSE: Mini Mental State Examination;

* Percentage of elderly participants with performance scores above or equal to the cut-off point based on education level (Brucki et al.25); CDT: Clock Drawing Test (Sunderland et al.26).

**Figure 1.**
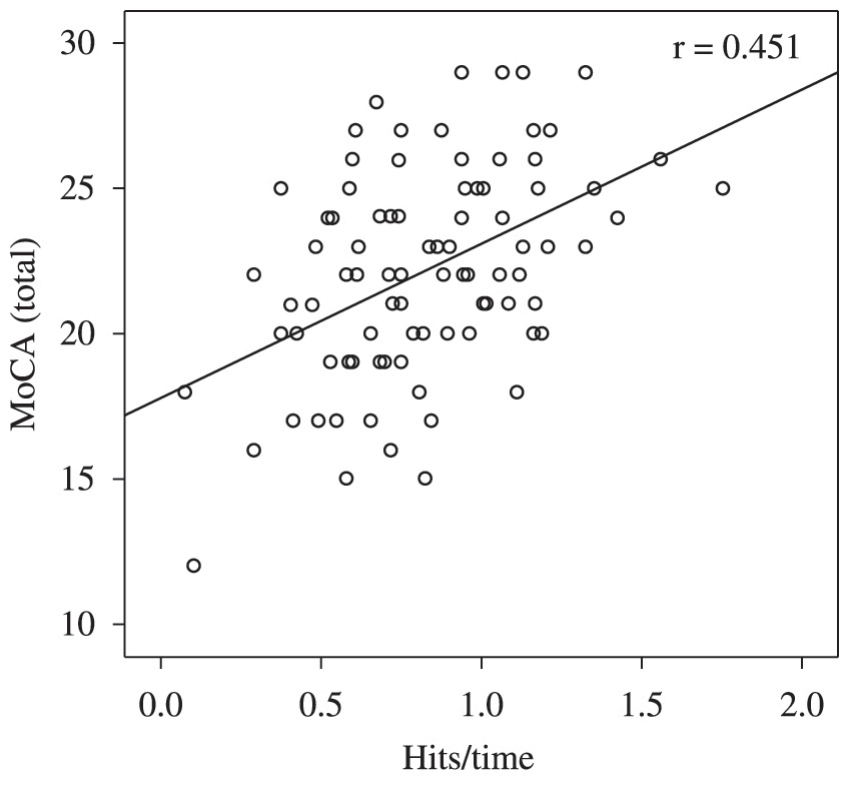
The relationship between the Montreal Cognitive Assessment (MoCA) total score and hits per unit of time of the Timed Up and Go - Dual Task (TUG-DT) for 98 subjects.

Table 3 describes the correlations between performance on the TUG-DT and the cognitive tests. Because the CDT and the MoCA domains orientation and abstraction were not correlated with any TUG-DT item (p>0.05), they were not included in Table 3. The visuospatial/executive function domain showed the strongest correlation with TUG-DT time (r=-0.339; p<0.01). In addition, the MoCA visuospatial/executive function domain exhibited significant correlations with the variables H/time (r=0.427; p<0.01) and words/time (r=0.336; p<0.01). The MoCA total score exhibited significant correlations with H/time (r=0.451; p<0.01; Figure 1) and words/time (r=0.340; p<0.01). The number of errors was correlated, albeit weakly, with the MMSE score (r=-0.271; p=0.01).

**Table 3.** Correlation between the Performance Scores Achieved on the Timed Up and Go - Dual Task (TUG-DT) Test and the Cognitive Tests for 98 Subjects.

Performance Criteria on TUG-DT Test	MoCA(total score)	Visuospatial/executive function	Attention	Naming	Language	Delayed recall	MMSE
Time	r=–0.227*	r=–0.339**	r=–0.212*	r=–0.227*	NS	NS	r=–0.313**
Hits	r=0.270**	NS	r=0.224*	NS	NS	NS	r=0.229*
Errors	r=–0.240*	r=–0.211*	r=–0.237*	NS	r=–0.184*	NS	r=–0.271**
Task cost	r=–0.213*	r=–0.217*	r=–0.240*	NS	r=–0.233*	NS	r=–0.285**
E/(H+E)	r=–0.252*	r=–0.220*	r=–0.254*	NS	r=–0.201*	NS	r=–0.289**
H/Time	r=0.451**	r=0.427**	r=–0.367**	NS	NS	r=0.209*	NS
Words/Time	r=0.340**	r=0.336**	r=–0.269**	NS	NS	NS	NS

TUG-DT: Timed Up and Go test with dual task; MoCA: Montreal Cognitive Assessment; MMSE: Mini Mental State Examination; E/(H+E) number of word errors per total words; H/time: hits per unit of time; Words/time: number of words per unit of time; NS: non-significance;

* p<0.05;

** p<0.01. Orientation and abstraction domains and the number of steps according to the TUG-DT are not included in the table because the correlations were not significant.

## Discussion

The present study investigated the correlation between TUG-DT performance and MoCA, CDT, and MMSE among older adults living in the community who exercise. Compared with the TUG cutoff point established for older Brazilian adults (12.47 seconds), the performance of the present sample on the TUG-DT was satisfactory: 9.56 seconds^22^. The median total MMSE score (28 points) matched the cutoff point established for older adults with 12 years of formal education (i.e., 29 - 1 standard deviation)^25^. In addition, the performance of 63.3% of participants was equal to or greater than the cutoff point adjusted for the number of years of formal education^25^. The performance of the sample with regard to the CDT was normal based on the expected scores of 9 and 10 among healthy older adults^26^.

Despite the satisfactory performance on the MMSE and CDT, the total MoCA score was less than the normal cutoff (i.e., 26) for 81.6% of the participants^24^. Satisfactory performances on the MMSE and CDT were expected because the assessed population was composed of independent individuals with high education levels who regularly exercised. However, their performance on the MoCA (an instrument designed to screen older adults for mild cognitive impairment) was poorer than expected, even though the participants were apparently healthy. This finding emphasizes the relevance of subjecting older adults to more thorough follow-up assessments, even when cognitive impairment is unlikely.

The adequate performance on the TUG-DT notwithstanding, this score was significantly correlated with the MoCA total score and the visuospatial/executive function domain. The establishment of cutoff points for dual-task tests and the inclusion of more challenging cognitive tasks are necessary for physical therapists to be able to quickly assess seemingly healthy populations who, nevertheless, might exhibit cognitive decline.

Previous studies^29^^-^ 32 have shown that executive function (one of the cognitive domains most affected by aging) is relevant for the performance of simultaneous tasks. This finding accounts for the strong relationship between performance on TUG-DT and executive function found in the present study. Theill et al.^33^ assessed the relationship between gait velocity and cognitive performance during single- and dual-task conditions, the latter involving working memory and semantic memory. The sample was primarily composed of men (average age=77 years old) without any deficit likely to impair their cognitive performance^33^. The results showed that participants' performances were poorer in the dual-task condition involving executive function (i.e. working memory)^33^. In the present study, however, CDT performance (which primarily assesses executive function) was not correlated with the TUG-DT variables. One possible explanation for this absence of correlation might be the narrow score range of CDT (1-10) and the choice of a cognitive task that was not challenging enough to test the executive function of the assessed population. The gait performances recorded during the dual-task tests were poorer than those recorded during the tasks demanding greater executive function and dynamic balance. Performance was also poor among individuals with associated depression^34^. Certain participant characteristics (e.g., depression, anxiety, and balance deficits) influence gait and mobility and might impair performance on dual tasks^34^. The participants in the present study exhibited satisfactory executive function and acceptable performance on the TUG . Most participants did not use psychotropic medications, their educational level was above the Brazilian average (7.4 years^1^), and most were non-fallers (86.7%). These characteristics might account for participants' satisfactory performances on the dual-task test^34^.

Hausdorff et al.^32^ investigated the influence of cognitive function on the dual-task performance of 711 older adults who were either healthy or who had cognitive impairments. The gait velocity of participants with cognitive impairment was slower for both the single and dual tasks (reciting the days of the week in reverse order starting from Sunday while standing up and walking), regardless of the cognitive task chosen for the latter. The groups did not differ with regard to the rate of errors in the cognitive task. The rate of cognitive errors was also low in the present study; however, the task cost revealed that participants' performances were poorer for the dual task than the single task. Other studies have shown that gait disorders are accentuated more among older adults with cognitive impairments and that such disorders become even more salient in dual-task situations^35^^-^ 38.

The meta-analysis performed by Al-Yahya et al.^39^ revealed significant gait deficits among older adults when the secondary task involved executive or memory functions (e.g., verbal fluency), but not when it was extremely simple (e.g., reaction time and discrimination tasks)^39^. Therefore, the gait performances of older adults seemed to deteriorate when they were combined with tasks that challenged executive function or memory^40^^-^ 42. Therefore, the relationship between dual-task and cognitive tests might depend on the difficulty that the assessed tasks posed to a particular population^43^.

Unlike previous research^12^^,^ 33, the present study not only assessed gait velocity but also the number of steps as well as variables related to the secondary task such as the number of hits per unit of time. The greatest correlations with the cognitive tests found in the present study were for the TUG-DT variables that have not usually been explored such as the numbers of hits per unit of time, errors per number of words, and words per unit of time. These variables could be easily analyzed by examiners.

The present study had limitations. The results could not be generalized to all older Brazilian adults living in the community because the sample was composed of adults who exercised regularly for at least 1 year and had higher levels of education. Nevertheless, cognitive assessments of this subpopulation of active and highly educated older adults (compared to Brazilian population) are highly relevant because (their profile notwithstanding) the performance of the participants on MoCA was below that expected. Importantly, the tests used in the present study are widely accessible and easy to apply in clinical practice. The TUG-DT task is quick, practical, inexpensive, and functional. Additional studies combining different types of cognitive tasks with the TUG task are needed to facilitate the detection of early cognitive decline among older adults who exercise. This detection will enable physical therapists to treat these individuals in a timely manner.

To summarize, the correlation between the TUG-DT results (i.e., time, hits/time, and words/time) and the MoCA visuospatial/executive function domain was weak. The strongest correlations with the MoCA were exhibited by the TUG-DT variables not usually considered such as the number of hits per unit of time. These findings suggest that the TUG-DT might be used in clinical practice as a functional and practical test for the early screening of cognitive dysfunction among older adults who exercise. However, additional studies including more challenging cognitive tasks in the TUG-DT task are needed to obtain stronger correlations with the cognitive tests.
